# Accessibility for people with motor disabilities at CEOs in Brazil: an ecological study

**DOI:** 10.1590/1807-3107bor-2025.vol39.080

**Published:** 2025-08-04

**Authors:** Djalma Antonio de LIMA, Magda Lyce Rodrigues CAMPOS, Elisa Miranda COSTA, Rejane Christine de Sousa QUEIROZ, Ana Margarida Melo NUNES, Nilcema FIGUEIREDO, Paulo Sávio Angeira de GOES, Erika Barbara Abreu Fonseca THOMAZ

**Affiliations:** (a) Universidade Federal do Maranhão - UFMA, Department of Dentistry, São Luís, MA, Brazil.; (b) Universidade Federal de Alagoas – UFAL, Department of Dentistry, Maceió, AL, Brazil.; (c) Universidade Federal do Maranhão - UFMA, Department of Public Health, São Luís, MA, Brazil;; (d) Universidade Federal de Pernambuco – UFPE, Department of Health Sciences Center, Recife, PE, Brazil.; (e) Universidade Federal de Pernambuco – UFPE Department of Clinical and Preventive Dentistry, Recife, PE, Brazil.

**Keywords:** Disabled Persons, Secondary Treatment, Latent Class Analysis, Architectural accessibility

## Abstract

The objective was to compare physical accessibility indicators for people with motor disabilities (PwMD) at dental specialty centers (acronym in Portuguese - CEO) across Brazil during the two evaluation cycles of the Access and Quality Improvement Program (acronym in Portuguese - PMAQ) for CEOs. This ecological study utilized secondary data from the external evaluation of PMAQ-CEO in Cycle I (C1), conducted in 2014, and Cycle II (C2), conducted in 2018, including all CEOs that participated in both cycles (n = 889). The structural items analyzed included corridors and doors adapted for wheelchairs, functional wheelchairs, access ramps with handrails, and bathrooms adapted for PwMD. Latent class transition analysis was applied to identify patterns in physical accessibility among CEOs, selecting the latent status (LS) model based on conceptual interpretability and goodness of fit. The final model identified five LS, labeled as follows: LS1 (most accessible); LS2 (inappropriate doors and bathrooms); LS3 (inappropriate ramps and bathrooms); LS4 (wheelchair unavailability); and LS5 (least accessible). In C1, 33.9% of the CEOs were highly accessible, while 17% were minimally accessible. In C2, these proportions shifted to 69.7% for highly accessible and 6% for minimally accessible. When analyzing the two PMAQ-CEO cycles, improvements in physical accessibility indicators were observed across Brazilian CEOs: corridors (9.7% increase) and doors (4.9%) adapted for wheelchairs; functional wheelchairs (15.7%); access ramps with handrails (38.7%); and bathrooms adapted for PwMD (19.6%). It may be concluded that physical barriers to PwMD in Brazilian CEOs were significantly reduced between 2014 and 2018, improving physical accessibility.

## Introduction

Brazil has more than 45 million people with disabilities (PwD), representing 23.92% of the population. Of this total, more than 13 million have physical disabilities.^1^ Among the types of physical disability, motor disability is the most prevalent and consists of a limitation in correctly executing movements, thereby restricting mobility.^2^ Consequently, policies aimed at expanding physical accessibility, including the health care network, are essential to safeguard the health rights of people with motor disabilities (PwMD). However, despite the legal framework in force since the Federal Constitution of 1988,^3^ physical barriers persist in the healthcare facilities of the Brazilian Public Health System (Sistema Único de Saúde, SUS). By law, PwD should have priority in collective and individual health actions. Nevertheless, despite their broad rights to healthcare access, inequities persist in practice,^4^ a concern that is particularly important in the context of developing countries.^5^

PwMD should have broad access to healthcare facilities, especially those designated for public use. Therefore, healthcare units should be free from physical barriers,^5^ including full physical accessibility at dental specialty centers (CEOs). These facilities should provide cost-free specialized dental services within the SUS framework, including at least the following specialties: oral diagnosis, focusing on oral cancer; specialized periodontics; minor oral surgery; endodontics; and care for PwD with motor, visual, or hearing impairments, as well as for people with special needs, such as pregnant women, people with hypertension, diabetes, heart disease, and other conditions.^6^

Considering the need to evaluate healthcare at CEOs, the Brazilian Ministry of Health launched the National Program for Improving Access and Quality at CEOs (PMAQ-CEO) in 2013 to foster advances in healthcare accessibility and in the quality of services offered throughout Brazil.^7,8^ Although microdata were in the public domain, few studies have utilized PMAQ-CEO data to evaluate accessibility issues for PwD.^9,10^ Few studies have addressed accessibility in healthcare facilities in general,^4,11-14^ and none have comparatively analyzed physical accessibility for PwMD in cycles 1 and 2 of the PMAQ-CEO.

Accordingly, the aim of this study was to assess the physical accessibility of Brazilian CEOs for PwMD, comparing two PMAQ-CEO cycles. It is hypothesized that there have been improvements in the physical accessibility of CEOs throughout cycles 1 (2013–2015) and 2 (2015–2019) of the PMAQ-CEO, benefiting PwD throughout the Brazilian territory. Thus, there were more corridors and doors adapted to accommodate wheelchairs, more wheelchairs were purchased, more access ramps with handrails were built, and spacious bathrooms were adequately adapted for PwMD. It is expected that this study will contribute to identifying priority areas for implementing strategies that directly benefit PwMD at CEOs.

## Methods

### Study design

This is a national ecological study using secondary data from the two PMAQ-CEO cycles, with CEOs as the units of analysis. The study followed the RECORD (REporting of studies Conducted using Observational Routinely collected health Data) extension to ensure a more robust methodological framework.

### Study location

Brazil is a large South American country with a territorial area of 8,516,000 km^2^. It is the world’s fifth largest country. With a Human Development Index (HDI) of 0.754 in 2021, Brazil ranked 87th among 191 countries. Currently, it has a population of 203 million inhabitants, distributed across 27 federative units (26 states and a Federal District),^1^ organized into five geopolitical macroregions: North (Population: 17,354,884; HDI: 0.667), Northeast (Population: 54,658,515; HDI: 0.663), Midwest (Population: 16,289,538; HDI: 0.757), South (Population: 29,937,706; HDI: 0.754), and Southeast (Population: 84,840,113; HDI: 0.766).^15^

The 1988 Federal Constitution of Brazil recognized health as a fundamental right for all citizens and a duty of the state, leading to the creation of SUS. This legal framework introduced a new political and organizational framework for health services and programs, also promoting measures to guarantee health protection and recovery, with funding provided on a tripartite basis by the federal, state and local governments.^16,17^ The doctrinal principles of SUS are universality, comprehensiveness, and equity.^18^ Services should be organized into hierarchical networks, with each municipality offering at least primary healthcare services. In contrast, health regions would offer medium- and high-complexity services (generally a consortium of several small towns).

### Study period and participants

Two periods of the PMAQ-CEO were analyzed: 2014 (cycle 1) and 2018 (cycle 2), considering the performance evaluations conducted during the external evaluation phase of the program. In 2014, the Brazilian Ministry of Health registered 988 CEOs;^19^ however, 932 (94.3%) were analyzed in cycle 1 of PMAQ-CEO.^20^ In 2018, the Brazilian Ministry of Health registered 1,097 CEOs^21^ and 1,042 (95%) were analyzed in cycle 2 of PMAQ-CEO.^8^ This study included only CEOs that participated in both cycles of the program, totaling 889 CEOs. The total number of CEOs assessed herein differs from that in the study by Queiroz et al.,^9^ given that this study analyzed fewer variables, allowing for the inclusion of more CEOs in the analysis. Conversely, the study by Queiroz et al.^9^ assessed 827 CEOs (in both PMAQ-CEO cycles).

Although participation in the PMAQ-CEO was voluntary in both cycles, there was satisfactory coverage of CEOs as a result of few refusals to join the program.^21^ Not all CEOs were evaluated in the two PMAQ-CEO cycles due to factors such as disqualifications of CEOs close to the evaluation period, inoperative CEOs due to renovation or maintenance, and problems accessing the facilities, which occur predominantly during rainy seasons in the northern and northeastern regions.^22^ Furthermore, the PMAQ-CEO was discontinued over the years due to political and partisan changes in the Brazilian federal government. Such changes, with fiscal austerity as a priority, led to institutional changes in the Brazilian Ministry of Health and public health policies. Primary healthcare funding was modified, and the program was discontinued.^23^

### Data collection process

The external evaluation phase of the PMAQ-CEO was coordinated and carried out by a consortium of universities, in partnership with the Brazilian Ministry of Health. Data were collected by trained dentists stationed at the CEOs. Data were collected through observation of the structure (module I), complemented by interviews with CEOs, managers, and professionals (module II), in addition to interviews with up to 10 users who were present at the CEOs at the time of the evaluators’ visit (module III).^24^

The study used data exclusively from module I of the PMAQ-CEO, which evaluated the infrastructure of the locations based on an observational script within the health units.^22^ PMAQ-CEO databases were collected in August 2022 on the program’s official website; however, all data from this program are no longer available for public access.

### Variables

The variables included in the study are described in Table 1, referring to questions about physical accessibility in the external evaluation of the PMAQ-CEO. The variables “corridors adapted for wheelchairs,” “doors adapted to accommodate wheelchairs,” “wheelchair, in usable condition, available for user movement,” “access ramp with handrail,” and “bathroom for users (adapted with a lower toilet, sink accessories, soap and paper dispensers at a lower level, support bars, outward-opening doors, and space for wheelchair maneuvering)” were considered in the latent transition analysis (LTA) - creating typologies of physical accessibility for CEOs according to these variables. It was verified whether there were changes in the classification (typology) of the CEOs between the cycles and whether these changes were related to the type of CEO, region, state and population size. A CEO was considered to have improved accessibility if it shifted from LS 5 in cycle 1 to any other LS in cycle 2 and from LS 2, 3, or 4 to LS 1.

**Table 1:** Description of study variables.

Cycle 1				Cycle 2			
Variable code	Variable description	Response categories		Variable code	Variable description	Response categories	
VII.5.1.1	Wheelchair-accessible corridors	1	Yes	I.4.1.1	Wheelchair-accessible corridors	1	Yes
		2	No			2	No
VII.5.1.2	Doors adapted to accommodate wheelchairs	1	Yes	I.4.1.2	Doors adapted to accommodate wheelchairs	1	Yes
		2	No			2	No
VII.5.1.3	Wheelchairs, in usable condition, available for user movement	1	Yes	I.4.1.3	Wheelchairs, in usable condition, available for user movement	1	Yes
		2	No			2	No
VII.5.1.4	Access ramp with handrail	1	Yes	I.4.1.4	Access ramp with handrail	1	Yes
		2	No			2	No
VII.9.1.3	Bathroom for users (adapted with a lower toilet, accessories with sink, soap and paper dispensers at a lower level, support bars, outward-opening doors, and space for wheelchair maneuvering)	1	Yes	I.8.1.3	Bathroom for users (adapted with a lower toilet, accessories with sink, soap and paper dispensers at a lower level, support bars, outward-opening doors, and space for wheelchair maneuvering)	1	Yes
		2	No			2	No
CEO_type	Typology of CEOs	1	Type 1	CEO_type	Typology of CEOs	1	Type 1
		2	Type 2			2	Type 2
		3	Type 3			3	Type 3
Region	CEO geographic region	1	North	region	CEO geographic region	1	North
		2	Northeast			2	Northeast
		3	Southeast			3	Southeast
		4	South			4	South
		5	Midwest			5	Midwest
FU	State, according to IBGE codes			FU	State, according to IBGE codes		

Source: PMAQ-CEO, Brazil. 2014 and 2018.

### Statistical analysis

A descriptive data analysis was carried out for both cycles, estimating absolute values, percentages, and 95% confidence intervals (95%CI). LTA was used to identify physical barriers for PwMD at CEOs across the two PMAQ-CEO cycles, modeling CEOs typologies (latent status-LS) and class transition over time.^25^ The probabilities of each CEO belonging to an LS were evaluated. Absolute frequencies and percentages of CEOs’ LS were also tabulated, estimating the proportional difference and change in LS between cycles 1 and 2 of the PMAQ-CEO. LTA considers that the units of analysis may change from one LS to another.^26^

Models with two to six LS were tested. The five-LS model was chosen, as it demonstrated the best concept and quality of fit: p-value > 0.05, according to the statistical likelihood ratio test (lowest chi-square), high values in the Akaike information criterion (AIC) and Bayesian information criterion (BIC) and entropy closest to 1. The chi-square test was used to analyze whether there was a difference in the proportion of CEOs that improved their accessibility status depending on the type of CEO, region, state, and population size (alpha = 5%). The analyses were performed using the MPlus software, version 8.4 (Muthen & Muthen, Los Angeles, USA), and Stata, version 17 (College Station, USA).

A choropleth map was generated for better visualization of the distribution of latent transition parameters related to the physical accessibility of CEOs in the Brazilian territory, incorporating the Brazilian Institute of Geography and Statistics data updated in 2019. The map was created with the QGIS program, version 3.30.0 (Chicago, USA).

### Ethical aspects

Secondary data obtained from the Brazilian Ministry of Health website for the PMAQ-CEO cycles were used. The census was approved by the Ethics Committee of the Federal University of Pernambuco (UFPE), process CAAE 23458213.0.0000.5208, on August 6, 2014 (cycle 1) and on January 30, 2018 (cycle 2), in compliance with the ethical resolutions of the Brazilian National Health Council. In this study, we did not use interview data; we only used data on the CEOs’ structure, which were collected in loco.

## Results

The comparison of the two PMAQ-CEO cycles revealed an improvement in the reduction of physical barriers at Brazilian CEOs for all analyzed indicators, including corridors and doors adapted for wheelchairs, suitable wheelchairs, access ramps with handrails, and bathrooms adapted for PwMD (Table 1). The greatest improvement across the cycles was observed in access ramps with handrails, whereas the least improvement was seen in doors adapted for wheelchairs.

Various models were tested with two, three, four, five, and six LS (Table 2). The five-LS model was selected, based on its adjustment properties (likelihood ratio chi-square; degrees of freedom; p-value; AIC; BIC; and entropy), as well as its superior conceptual cohesion and alignment with the physical accessibility characteristics of CEOs, considering the physical barriers outlined in Table 1.

**Table 2:** Physical accessibility characteristics of dental specialty centers for people with motor disabilities, comparing the two PMAQ-CEO cycles.

Structural features	Cycle 1	Cycle 2	p-value
	n (%)	n (%)	
Corridors adapted for wheelchairs			<.001
Yes	678 (76.2)	770 (86.6)	
No	211 (23.8)	119 (13.4)	
Doors adapted for wheelchairs			<.001
Yes	695 (78.1)	749 (84.2)	
No	194 (21.9)	140 (15.8)	
Suitable wheelchairs			<.001
Yes	541 (60.8)	676 (76.0)	
No	348 (39.2)	213 (24.0)	
Access ramps with handrails			<.001
Yes	423 (47.6)	773 (86.9)	
No	466 (52.4)	116 (13.1)	
Bathrooms adapted for people with special needs			<.001
Yes	405 (45.6)	590 (66.3)	
No	484 (54.4)	299 (33.7)	

Source: PMAQ-CEO, Brazil. 2014 and 2018.

Table 3 presents the five-LS model of physical barriers at CEOs. LS1 was the most accessible model with high accessibility, but it required an increase in adapted bathrooms (75.9%). In LS2, most CEOs had high accessibility with adapted ramps (89.3%), but they required an increase in adapted doors (61.1%) and bathrooms (52.7%). LS3 showed an opposite trend, with good accessibility for the use of adapted doors (91.9%) but required an increase in ramps (84.6%) and bathrooms (59.4%) adapted for PwMD. In LS4, CEOs did not have wheelchair accessibility (100%). In LS5, CEOs had low accessibility for the five structure items analyzed.

**Table 3:** Adjustment characteristics of the five latent transition analysis models.

	Number of latent classes	Likelihood ratio chi-squarea	Degrees of freedom	p-value^b^	AIC^c^	BIC^d^	Entropy^e^
CEO ambience	2	1065.08	1010	> 0.999	8882.02	8944.29	0.763
	3	741.78	1000	> 0.999	8578.72	8688.89	0.781
	4	659.24	987	> 0.999	8531.31	8698.96	0.722
	5	588.85	973	> 0.999	8488.17	8722.89	0.785
	6	530.06	957	> 0.999	8461.05	8772.41	0.775

^a^Likelihood ratio chi-square. Reference value: low; ^b^p-value reference value: > 0.05; ^c^AIC: Akaike Information Criterion. Reference value: high; ^d^BIC: Bayesian Information Criterion. reference value: high;^e^ Entropy reference value: close to 1.0.

Source: Authors.

In PMAQ-CEO cycles 1 and 2, the proportion of CEOs in LS1 (more accessible) increased by 105.6% (33.9–69.7); LS2 (improper doors and bathrooms) increased by 279% (2.9%–11.0%); LS3 (improper ramps and bathrooms) decreased by 98.6% (34.9%–0.5%); LS4 (wheelchair accessibility problems) increased by 13.6% (11.0%–12.5%); and LS5 (least accessible) decreased by 64.7% (17.0%–6.0%) (Table 3).

There was a high probability of CEOs remaining in LS1 (89.7% - more accessible) - and in LS4 (80.6% - wheelchair accessibility problems); and a low probability of remaining in LS5 (19.1% - less accessible); LS2 (3.8% - improper doors and bathrooms) and LS3 (0.3% - improper ramps and bathrooms) (Table 3). Almost half of the CEOs improved their LS between the cycles (n = 413; 46.46%). The shift from an LS with worse accessibility to an LS with better accessibility was not related to the type of CEO, region, state and population size (p > 0,05) (data not shown).

Roraima (RR) (100%, n = 1), in the north, was the state with the highest proportion of CEOs moving from a less accessible to a more accessible LS. However, this state had a single CEO, and therefore, any change would represent 100%, followed by Tocantins (TO) (71.4%, n = 5), Paraíba (PB) (64.1%, n=34), Sergipe (SE) (62.5%, n = 5), and Paraná (PR) (61.4%, n = 27). On the other hand, the Federal District (FD) (0%), in the midwestern region, showed the lowest proportion of improvement, followed by the states of Minas Gerais (MG) (33.7%, n = 26), Pernambuco (PE) (35.9%, n = 14), Pará (PA) (37.0%, n = 10), Espírito Santo (ES) (37.5%, n = 3), Santa Catarina (SC) (39, 5%, n = 17), and Maranhão (MA) (40.0%, n = 10) (Figure).

**Figure:**
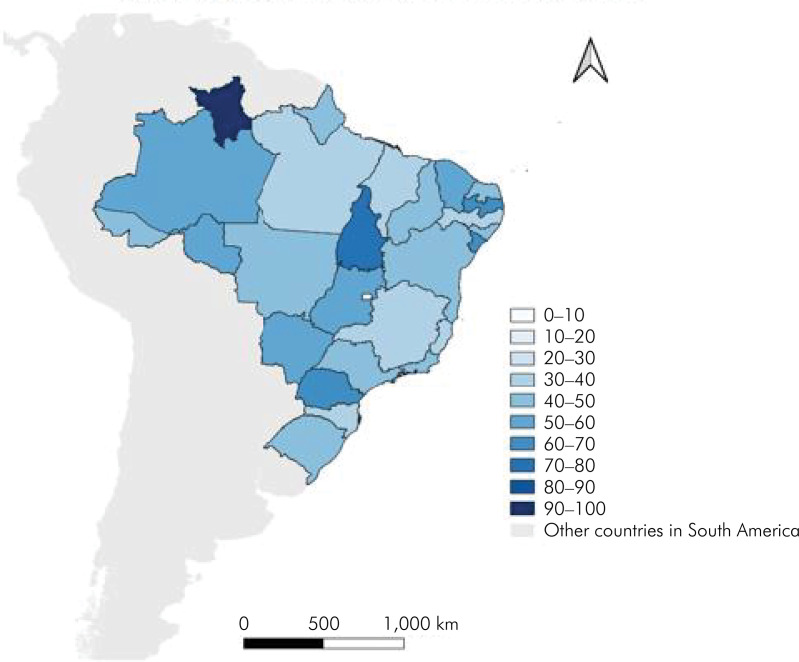
Choropleth map of the proportion of change in latent status (less accessible to more accessible) of physical accessibility of Dental Special Centers, by federative unit. Regarding the blue tones on the map, the darkest blue was where it more improved and the lightest blue was where it less improved.

## Discussion

Comparison of the two PMAQ-CEO cycles revealed positive changes in the physical accessibility of CEOs concerning physical barriers that can hinder full patient access to the health unit. This study consists of the first ecological temporal analysisof the physical barriers faced by PwMD at Brazilian CEOs.

LTA allowed identifying and comparing the changes at CEOs in the two PMAQ-CEO cycles. The models were developed based on five physical accessibility variables in the health unit, and the one with the best fit formed five LS through the LTA, namely: LS1 (more accessible), LS2 (improper doors and bathrooms), LS3 (improper ramps and toilets), LS4 (wheelchair accessibility problems), and LS5 (less accessible). The major transitions involved the migration of LS2 and LS3 to LS1 and the permanence of CEOs in LS4 and the migration of CEOs from LS5 to more accessible categories for PwMD (Table 4).

**Table 4:** Five-class model of physical barriers at dental specialty centers.

Latent class transition steps	Latent Status				
	Class 1	Class 2	Class 3	Class 4	Class 5
	More accessible	Inappropriate doors and bathrooms	Inappropriate ramps and bathrooms	Wheelchair accessibility issues	Less accessible
Item response probability (latent class/status)					
Corridors adapted at CEOs					
Yes	100.0	51.2	89.3	96.3	6.2
No	0.0	48.8	10.7	3.7	93.8
Adapted doors at CEOs					
Yes	100.0	38.9	91.9	92.4	9.9
No	0.0	61.1	8.1	7.6	90.1
Wheelchairs at CEOs					
Yes	92.6	73.9	67.2	0.0	25.1
No	7.4	26.1	32.8	100.0	74.9
Adapted ramps at CEOs					
Yes	94.0	89.3	15.4	66.0	15.2
No	6.0	10.7	84.6	34.0	80.8
Adapted bathrooms at CEOs					
Yes	75.9	47.3	40.6	50.8	13.1
No	24.1	52.7	59.4	49.2	86.9
Latent status ratio					
Time 1 (PMAQ-CEO cycle 1, 2014)	33.9	2.9	34.9	11.0	17.0
Time 2 (PMAQ-CEO cycle 2, 2018)	69.7	11.0	0.5	12.5	6.0
Transition probabilities					
More accessible	89.7	9.6	0.0	0.0	0.7
Inappropriate doors and bathrooms	96.2	3.8	0.0	0.0	0.0
Inappropriate ramps and bathrooms	85.2	7.7	0.3	0.0	6.8
Wheelchair accessibility issues	0.0	17.4	0.0	80.6	2.0
Less accessible	38.8	17.8	2.6	21.7	19.1

Physical barriers directly interfere with the free movement of patients, especially for PwMD, generating significant difficulties. Inappropriate ramps, stairs, high steps, and bathrooms without appropriate adaptations for PwMD are examples of the structural challenge these people face in many healthcare settings.^14^ In LS5 CEOs, among the five variables analyzed, there is a high percentage of inappropriate physical accessibility, showing that, historically, the inclusion of accessibility aspects was not always prioritized in their construction.

These barriers directly affect the quality of care provided and patient experience, especially for those with upper limb motor impairments.^27^ The lack of accessibility can impact the ability of these individuals to perform daily activities, such as maintaining proper oral hygiene, thus increasing their vulnerability to oral diseases.^28^ In addition, the lack of physical accessibility limits the autonomy of these individuals, generating greater dependence and decreasing their well-being in the healthcare environment.

If it is impossible to provide PwD with dental care in the primary care setting, these patients are referred to CEOs. These are generally more complex patients, requiring considerable physical accessibility. Therefore, it is crucial to have appropriate infrastructure at CEOs, providing safety and well-being to patients with some disability or reduced mobility,^20^facilitating the movement of patients within the health unit.

The findings of this study show that structural flaws are not limited to difficulties in movement but also directly impact equity in access to oral healthcare. By failing to provide an inappropriate infrastructure, such as ramps, adapted bathrooms, and accessible spaces, health units exclude patients with PwD, reinforcing inequalities. More accessible CEOs, such as those in LS1, are examples of spaces where existing adaptations facilitate patient reception and promote more inclusive and respectful care. However, improvements are still needed, particularly in aspects such as the adequacy of bathrooms for PwMD.

Therefore, the impact of lack of accessibility goes beyond physical barriers and immediate patient safety because it affects patients’ quality of life, emotional well-being, and even the success of dental treatments. The presence of accessible structures allows for greater autonomy, reduces dependence on third parties, and provides a more inclusive and efficient care environment.^20^ By following technical standards and implementing items such as appropriate ramps, non-slip floors, and handrails, health units prevent accidents and increase the autonomy of PwMD,^29^ fostering respect for the dignity and individuality of each patient. Thus, accessibility should be understood as a fundamental element for inclusion, ensuring that everyone can access health services under equal conditions, regardless of their physical limitations.

CEOs are registered under a specific ordinance and should ensure proper physical accessibility to receive all patients, including PwMD.^30^ Therefore, the NBR 9050 standards were first published in 1985, and since then, they have gone through four revisions: in 1994, 2004, 2015, and 2020, with its improved version released in 2021. In its latest version (NBR 9050/2020), more criteria and technical parameters were established to guide the design, construction, installation, and adaptation of urban and rural environments and buildings, ensuring compliance with accessibility standards. This standard does not replace the laws, decrees, or regulations that technical managers must comply with when preparing their projects.^31^ While this standard defines accessibility requirements for health units, such as CEOs, its enforcement has been inconsistent in practice. Many CEOs present different types of physical barriers,^27^ as evidenced by LS2, LS3, LS4, and LS5, all of which exhibit deficiencies in one or more physical accessibility features.

A study analyzed the accessibility of health services in São Paulo (Brazil) for 25 people with some type of disability (limb paralysis or amputation; low vision, unilateral or total blindness; low hearing, or unilateral or total deafness). The authors evaluated the following criteria: time of service; availability of easy parking; presence or absence of ramps; number of available seats in the waiting room; easy access to the room; availability of usable wheelchairs; presence of truly qualified professionals; and existence of proper signage. All interviewees indicated that they had some difficulty related to the analyzed variables.^32^ Compared to the present study, that study evaluated more variables, allowing for a more comprehensive view of accessibility, ranging from the time of care to the presence of specialized professionals to treat PwD.

Many CEOs (73.2%) maintain headquarters, allowing managers to improve accessibility by removing physical barriers. This is especially important because CEOs are a reference for providing care to PwD.^10^ Nevertheless, despite financial incentive policies, only one-third of CEOs allocate 40 hours per week to the clinical care of PwD.^33^

Analysis of cycles 1 and 2 of the PMAQ-CEO showed an increase in the number of CEOs that provide proper physical accessibility for PwMD. Enhanced removal of physical barriers allows CEOs to achieve better monthly production goals.^33^ This study, however, did not provide an in-depth evaluation of the physical barriers faced by PwMD at CEOs, but it did examine hospital care and the assurance of treatment delivery. Improvements in accessibility were not assessed in terms of CEO size, states, geopolitical regions, or the population size of the municipality in which each CEO operates. The observed changes may be influenced by the political context, which can either facilitate or hinder the allocation of financial resources required for structural improvements.

Queiroz et al.^9^ analyzed whether there were improvements in care for people with some disability, comparing cycles 1 and 2 of the PMAQ-CEO. They found improvements in care for these users between 2014 and 2018, which contributed to strengthening the oral health of PwMD.^9^ However, their study did not focus on physical barriers faced by PwMD and did not incorporate the variables included in the present study. Instead, the study used other indicators, such as the number of dentists treating PwD and their weekly workload. Moreover, the authors did not find significant differences in the proportion of CEOs that improved accessibility based on CEO type, local human development index, or population size.^9^

A nationwide study conducted in 2017 analyzed the capacity of Brazilian CEOs to provide care for PwD. Of the 133 CEOs evaluated, only 79 had appropriate physical accessibility for PwMD. The midwestern region presented the highest provision of care for these individuals at CEOs (87.5%), whereas the southern region had the lowest provision (11.1%).^20^ According to the choropleth map, the midwestern and southern regions changed from a less accessible LS to a more accessible LS, showing a reduction in physical barriers. However, these data are still a cause for concern, given that the most accessible region fails to reach 90%. The southern region, which is more well-developed, should be expected to present a higher compliance with accessibility requirements.

The findings from the choropleth map indicate that, when comparing the two PMAQ-CEO cycles, the highest proportion of CEOs that improved their LS was observed in the state of Roraima (RR) (100%). Considering that RR had a single CEO, any improvement would represent all CEOs; therefore, this result should be viewed with caution. We suggest that further studies be carried out, focusing on the state of RR, and that an in-depth analysis be made of the improvements and challenges in the physical accessibility of CEOs. In addition to RR, all other states in the north, except for PA, and four of the nine states in the northeastern region (CE, PB, AL, and SE) showed improvements in 50% or more of CEOs, which shifted from a less accessible to a more accessible LS. These improvements may reflect public policies aimed at expanding access and reducing health inequities during this period, mainly focused on the northern and northeastern regions.^34,35^

A decade-long analysis (from 2010 to 2019) of the characteristics of CEOs revealed slight improvements in physical accessibility for PwMD, as well as in specialized oral healthcare.^10^ However, further improvements can be obtained in the regional distribution of CEOs (considering local and regional differences),^33^ in addition to the removal of physical barriers within health facilities for the promotion of universal accessibility.^10^

Most CEOs are located in municipalities with a small population size (64.2%), studies indicate that the larger the population or growth, the better the physical accessibility of CEOs.^28,36^ CEOs should be strategically distributed to meet the demand of health regions and they should preferably be located in the region’s main municipalities, thereby improving access to services.

The strengths of this work lie in its national scope, which allows for the identification of changes at CEOs across nearly all healthcare facilities operating in Brazil; the low risk of measurement bias, considering that the data were collected during an on-site visit by previously trained evaluators; the novelty of the findings regarding physical barriers faced by PwMD; the use of LTA, a robust approach with great potential to support the assessment of healthcare facilities and the identification of typologies that may change over time; and identification of priority areas on a choropleth map, with their categorization into states and macroregions.

A limitation of this study was the impossibility to analyze inoperative CEOs or those implemented after the first PMAQ-CEO cycle, based on a methodological decision, as the study focused on comparing the two PMAQ-CEO cycles. For such comparison, the same CEOs should be present in both cycles. Furthermore, the analysis was restricted to structural aspects related to PwMD. Other studies with different approaches (including others disabilities) are recommended. It has not been documented whether the three types of CEOs (I, II, and III) differ in terms of physical accessibility, as they may present different types and potentially different financial contributions. One of the limitations is the fact that some states, such as RR, have a small number of CEOs; therefore, any change represents a huge percentage, which could lead to an overestimation of changes in physical accessibility.

Furthermore, there has been a decline in the number of CEO managers who work only in health unit management. These professionals tend to also work in clinical care. This can negatively influence the planning, organization, and management of CEOs, including the identification of physical barriers, and may limit the development of public policies aimed at improving the accessibility of CEOs for PwD.^37^ In the absence of a dedicated manager, CEOs may rely on individuals without the necessary qualifications performing this role, which may interfere with the quality of management, structure, and services provided.^10^ Therefore, further studies should be carried out, including more variables, such as CEO types and the presence or absence of an on-site manager.

## Conclusion

An improvement in the physical accessibility of CEOs was observed in the PMAQ-CEO cycles, with more appropriate adaptations made for wheelchair users and PwMD. Significant progress was made in the adaptation of corridors and doors, the acquisition of appropriate wheelchairs, and the construction of ramps with handrails, with ramps being the most improved aspect. On the other hand, adapted doors for wheelchairs showed the least improvement, and adapted bathrooms for PwMD had the lowest percentage of adequacy at the end of cycle 2, indicating some ongoing need for improvement. Therefore, implementing strategies aimed at expanding door and bathroom adaptations, along with the maintenance of ramps and wheelchairs, should be a priority to ensure greater accessibility and comfort for PwMD.

## Data Availability

The authors declare that all data generated or analyzed during this study are included in this published article.
